# Structure-Based Design of Potent and Selective Ligands at the Four Adenosine Receptors

**DOI:** 10.3390/molecules22111945

**Published:** 2017-11-10

**Authors:** Willem Jespers, Ana Oliveira, Rubén Prieto-Díaz, María Majellaro, Johan Åqvist, Eddy Sotelo, Hugo Gutiérrez-de-Terán

**Affiliations:** 1 Department of Cell and Molecular Biology, Uppsala University, Biomedical Centre (BMC), BOX 596, SE-751 24 Uppsala, Sweden; willem.jespers@icm.uu.se (W.J.); analuisa.novodeoliveira@icm.uu.se (A.O.); johan.aqvist@icm.uu.se (J.Å.); 2 Centro Singular Investigación Quimica Biologica e Materiales Moleculares (CIQUS), Departamento de Quimica Orgánica, Facultade de Farmacia, Universidade de Santiago de Compostela, 15782 Santiago de Compostela, Spain; rupridi@gmail.com (R.P.-D.); maria.majellaro@uniba.it (M.M.); e.sotelo@usc.es (E.S.)

**Keywords:** free energy perturbation (FEP), G protein-coupled receptors (GPCRs), molecular dynamics (MD) simulations, structure-based drug design (SBDD)

## Abstract

The four receptors that signal for adenosine, A_1_, A_2A_, A_2B_ and A_3_ ARs, belong to the superfamily of G protein-coupled receptors (GPCRs). They mediate a number of (patho)physiological functions and have attracted the interest of the biopharmaceutical sector for decades as potential drug targets. The many crystal structures of the A_2A_, and lately the A_1_ ARs, allow for the use of advanced computational, structure-based ligand design methodologies. Over the last decade, we have assessed the efficient synthesis of novel ligands specifically addressed to each of the four ARs. We herein review and update the results of this program with particular focus on molecular dynamics (MD) and free energy perturbation (FEP) protocols. The first in silico mutagenesis on the A_1_AR here reported allows understanding the specificity and high affinity of the xanthine-antagonist 8-Cyclopentyl-1,3-dipropylxanthine (DPCPX). On the A_2A_AR, we demonstrate how FEP simulations can distinguish the conformational selectivity of a recent series of partial agonists. These novel results are complemented with the revision of the first series of enantiospecific antagonists on the A_2B_AR, and the use of FEP as a tool for bioisosteric design on the A_3_AR.

## 1. Introduction

The superfamily of G protein-coupled receptors (GPCRs), which encompasses targets for more than 30% of marketed drugs [1], was traditionally relegated from the field of structure-based drug design (SBDD) due to the inherent difficulty of solving the structure of these membrane receptors. However, recent advancements in membrane crystallography in the past decade have led to an explosive growth in available crystal structures, currently comprising 196 receptor–ligand complexes [2]. A particularly privileged family in this sense is the adenosine receptors (ARs), with several complexes of the A_2A_ and lately for the A_1_ ARs deposited in the PDB. These structures can be used to model the remaining A_2B_ and A_3_ receptor subtypes in the family, thus providing a full pallet of atomistic models of the AR family. ARs play an essential role in many physiological processes in the cardiovascular and central nervous systems, and control anti-inflammatory and immunosuppressive responses. Consequently, this family of receptors is of outstanding interest as drug targets for cardiovascular, neurodegenerative and autoimmune diseases, as well as cancer [3].

Over the last decades, a large number of agonists and antagonists have been synthetized and pharmacologically characterized, accumulating to 25,000 entries in the ChEMBL database, which have been source of a number of computational screening and SAR analyses [4,5,6,7]. The exponential growth of experimental GPCR structures [8] proved extremely beneficial for the structural biology and structure-based ligand design of ARs [9,10]. The current repertoire, consisting of several A_2A_AR structures in complex with antagonists and agonists, was recently complemented with fully active conformation of this receptor in ternary complex with a G protein mimic [11], and two antagonist-bound (inactive) A_1_AR structures [12,13]. The effects of point mutations on ligand binding constitute another important information resource for the characterization of receptor–ligand interactions and receptor activation. Thus, site-directed mutagenesis data further complement the structural and chemical information, and over 2500 data points are deposited in the GPCRdb [2]. This combination of structural, pharmacological and chemical information allowed the pharmaceutical SBDD of 1,2,4 triazines as A_2A_AR antagonists, one of which reached clinical studies for the treatment of Parkinson’s disease [9].

The family of ARs, and in particular the A_2A_AR, have become targets for several computational studies to evaluate methods and protocols proposed in the field of GPCR ligand design [10]. One of the first topics investigated after the release of the first A_2A_AR structure was the actual role of water molecules in ligand binding, studied through molecular dynamics (MD) sampling of the solvent in the binding cavity [14]. This allowed the identification of water clusters whose displacement should favor the binding affinity of prospective ligands [15], or of structural waters that should be considered for antagonist docking and virtual screening (VS) on this receptor [16]. The detailed structural characterization of the A_2A_AR also allowed for the estimation of binding free energies, based on MD sampling. The low affinity of the micromolar antagonist caffeine was rationalized using the molecular mechanics/Poison-Boltzman surface area (MM/PBSA) method [17], introducing the idea of a multiple binding mode for this molecule, which was partially supported by recent crystal structures with xanthine-like antagonists [13,18]. In this area, our group developed a protocol based on free energy perturbation (FEP) for the estimation of relative binding affinities upon point mutations [19]. The results of this “in-silico” site directed mutagenesis can be directly compared to experimentally determined ligand affinity ratios between wild-type (WT) and mutant receptor variants, coming from the numerous site-directed mutagenesis studies as we will discuss here for the case of the A_2A_AR [20,21]. The method, which was initially developed and later applied in other GPCR systems [19,22], overcomes the convergence problems associated with large perturbations and allows reproducing the effect of point mutations on ligand binding with high precision and convergence.

**Figure 1:**
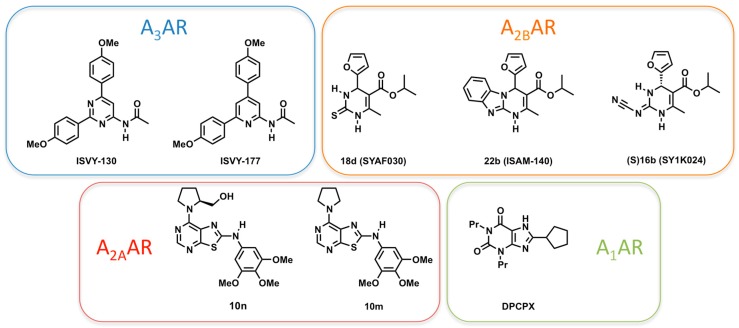
Adenosine receptor antagonists discussed in this work.

During the last years, our labs have combined FEP and other computational methodologies with high-throughput synthetic methodologies and pharmacological characterization, to develop various series of structurally simple, novel AR ligands [23]. Our SBDD protocol includes homology modeling, protein–ligand docking, 3D-QSAR, MD and FEP simulations, and was used to assist the design of novel ligands as well as to predict or rationalize their pharmacological profile [24,25,26,27,28,29,30,31,32]. The different series obtained with this strategy, represented in Figure 1, have been optimized to yield potent and selective antagonists of the different ARs. The first scaffold reported, a 2,4-diarylpyrimidine, was recently followed by a bioisosteric series of 2,4-diarylpyridines, both of them revealing high affinity and selectivity as A_3_AR antagonists [24,25,30]. For the A_2B_ receptor, we have documented the first non-planar, enantioselective antagonists, and disclosed the reasons of their enantiospecificity [31,32]. The A_2A_AR emerges as the most attractive target for SBDD within the AR family, due to the existence of several crystal structures complemented with broad mutagenesis and SAR data. Consequently, we used this system to train our protocol for in-silico mutagenesis, based on well-converged FEP calculations [20,21]. Moreover, we went one step further in the design of new ligands and isolated, from a series of antagonists, a group of compounds that presented a pharmacological profile of partial agonists, where we identified a prolinol moiety as a replacement of the ribose group of classical agonists [28]. Finally, recently published crystal structures of the A_1_AR allow for the characterization of molecular determinants of high affinity, which we here exemplify with the calculation and interpretation of the effect of point mutations on the binding affinity of the reference xanthine-like antagonist DPCPX (see Figure 1).

In the following sections, we review our recent results on the design and characterization of ligand binding for each of the four ARs, and further complement these studies with new analysis and calculations. We put the results of this project in the broad perspective of ARs ligand design, and discuss the methodological developments in our SBDD pipeline, with an impact in the design of novel ligands for this and other families of receptors.

## 2. Results

### 2.1. A_2A_AR

The A_2A_AR is one of the best-characterized GPCRs, with various entries in the PDB including inactive structures, in complex with eight different antagonists, four active-like structures with agonists, and one fully-activated A_2A_AR, in a ternary complex with an agonist and a mimic of the intracellular G protein [11]. In addition, this receptor accumulates 42% of the mutagenesis data deposited in the GPCRdb for the ARs, while 31% of the 26.274 entries in ChEMBL [33] (release 23) report affinities for this receptor subtype. The vast amount of chemical and structural information corresponds to the biopharmaceutical interest in this receptor, where antagonists have reached clinical trials as a nondopaminergic therapy for Parkinson’s disease [34], while the agonist Regadenoson has been approved by the FDA as a coronary vasodilator [35].

Given this unique combination of mutagenesis and structural information, we have used this system to further develop our in silico mutagenesis approach based on FEP [20,21]. The thermodynamic cycle underlying this approach was originally developed by Kollman and co-workers to characterize the effect of a single mutation on ligand binding free energies (and catalysis) [36], and consists on the perturbation of the amino acid sidechain from WT to mutant in both the complex and the apo versions of the receptor (which acts therefore as the reference state). Starting from this concept, we developed a dedicated FEP protocol to routinely evaluate affinity shifts for a given ligand to mutant versions of the receptor, as compared to the WT. Although standard FEP implementations might work well enough for simple cases (e.g., Serine to Alanine), more drastic mutations (e.g., Tryptophan to Alanine) are subject to convergence and sampling problems, usually leading to high hysteresis values (defined as the difference in the forward and backwards pathway of the sidechain transformation). In our implementation, described in detail in the methods section and applied here to the original characterization of mutagenesis experiments on the A_1_AR (see below), the sidechain of the residue to mutate is gradually annihilated to alanine in a stepwise fashion. With this strategy, we examined the effect of 18 alanine mutations reported for the A_2A_AR for agonist and antagonist binding with high precision (s.e.m. = 0.7 kcal/mol) and convergence values (average hysteresis of 0.2 kcal/mol) [21]. This analysis revealed effects that were not evident by the sole analysis of crystal structures, such as the disruption of water-mediated interactions causing decreased antagonist binding observed in mutants V84^3.32^A, L294^6.51^A or Ile274^7.39^A (Ballesteros–Weinstein nomenclature for GPCRs is indicated in superscripts as X.YY, with X indicating the helix and YY the correlative position relative to the most conserved residue in the helix, when YY = 50 [37]). Another outcome of such analysis was deciphering the detrimental effect on agonist binding of the M177^5.38^A mutation due to an increased solvation of the binding site that perturbed agonist-receptor interactions. The methodology was then extended to account for non-alanine mutations, by joining two thermodynamic cycles describing the reduction of a sidechain, i.e., from WT and mutant, respectively, to a common fragment (e.g., Alanine). This allowed us to further characterize the effect of 17 non-alanine mutations on agonist binding, again in excellent agreement with the experimental data [20].

**Figure 2:**
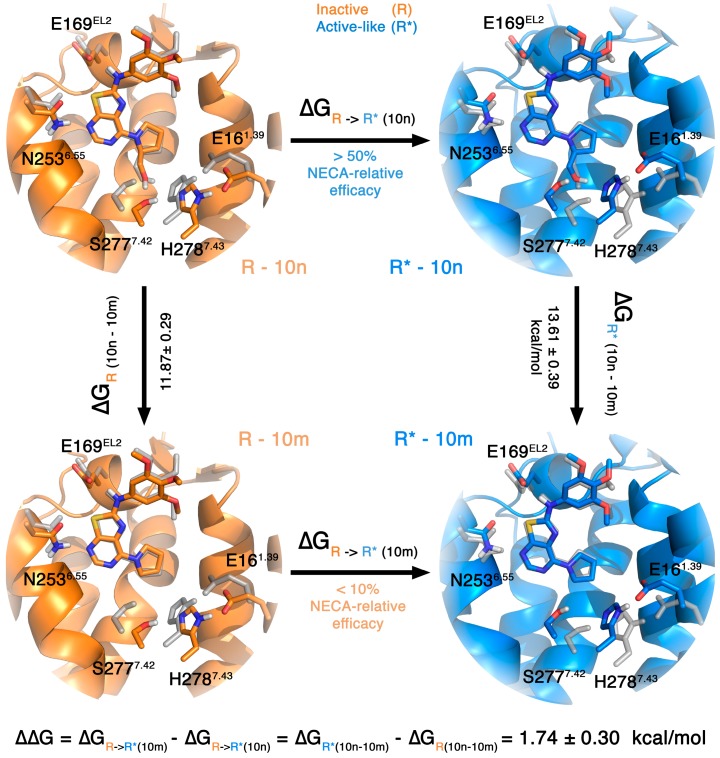
Thermodynamic cycle showing the relationship between the potency of the ligand pair **10m**/**10n** and the relative free energies of perturbing **10n** to **10m** in the active-like (blue) and inactive (orange) structures. The grey sticks represent the conformation of the residues in the active-like structure as compared to the inactive structure, and vice versa. A ligand that activates the receptor (partial or full agonist) should in theory have a higher affinity for the active state of that receptor. Thus, perturbing the (partial) agonist (**10n**) into an antagonist (**10m**) should result in an unfavorable change in binding free energies, as shown here for the **10n**→**10m** transformation.

The most traditional and extensive use of FEP calculations on ligand design, however, is to predict the relative binding affinity between pairs of ligands. In this case, the application involves a thermodynamic cycle where ligand 1 is transformed into ligand 2 in both the bound and the reference (i.e., water solvated) states. An example of the application of this technique on antagonist design can be found in the section covering the A_3_AR (see below). We herein decided to mix the idea of a ligand-perturbation with a receptor-comparison in a new thermodynamic cycle, which we apply to identify the preferred receptor conformational state for different pharmacological classes of ligands. In theory, the capacity of a ligand to activate the receptor (i.e., agonist potency) can be related to its specific preference for the active conformation. Therefore, the relative affinities between two ligands for the inactive and active conformations of the receptor can be qualitatively related to their relative difference in potency. To illustrate this point, we here perform such calculations on our recently reported series of thiazolo[5,4-*d*]pirimidines, designed as potential agonists of the A_2A_AR as bioisosteres of the purine ring on the basis of molecular docking and superposition with the classical agonists (e.g., adenosine, NECA) [28]. The molecules in this series that contain a 7-prolinol substitution were characterized as a new class of non-nucleoside A_2A_AR partial agonists, confirming the hypothesis of the design where the 2-hydroxymethylene moiety of the prolinol would mimic the interactions of the 2′/3′ hydroxyl groups of the ribose of agonists, observed in all agonist bound A_2A_AR crystal structures. To understand the molecular determinants of the functional behavior of these molecules, we designed a thermodynamic cycle where the partial agonist ligand **10n** (containing prolinol, relative efficacy as compared to NECA > 50%) was transformed in its antagonist analog lacking the 2-hydroxymethylene group (**10m**, with a relative efficacy value < 10%), both in the inactive and active-like A_2A_AR structures as shown in Figure 2. The results indicate that the loss of the 2-hydroxymethylene “agonist tag” is more unfavorable in the active-like than in the inactive receptor structure (∆∆*G***_10m→10n_** = 1.74 ± 0.29 kcal/mol), which is in qualitative agreement with the observed reduction in potency between this pair of compounds. Interestingly, the main difference in the simulations between the inactive and active structures are observed in the interactions of the 2-hydroxymethylene moiety, where the interaction with S277^7.42^ in the (unfavored) inactive conformation is replaced by a hydrogen bond to H278^7.43^ in the simulations of the active-like structure. This might explain why there are several partial agonists that exert a higher potency on the S277A^7.42^ mutant receptor [38]. If followed a similar evaluation of a second pair of compounds within this series with similar results: The partial agonist denoted as **10d** (40% efficacy as compared to NECA) contains a different substitution pattern on the ring exposed to the extracellular loops (i.e., 4-Cl-phenyl instead of 3,4,5-tris-OMe-phenyl in **10m**/**10n**, see Figure 1), and is transformed into the corresponding antagonist (**10c**, 10% efficacy) upon removal of the prolinol moiety. The calculated effect in this pair of ligands also indicates that the affinity of the partial agonist for the active form of the receptor is favored as compared to the antagonist. Moreover, the reduced energetic barrier as compared to the **10m**/**10n** pair (∆∆*G***_10d→10c_** = 0.65 ± 0.26 kcal/mol) agrees with the lower experimental difference in the efficacy between the **10d**/**10c** pair of compounds.

### 2.2. A_1_AR

The adenosine A_1_ receptor, which plays a significant role in the regulation of neural, renal and cardiac systems [3], was the first AR to be characterized [39]. A_1_AR antagonists have been predominantly derivatives of the xanthine scaffold, such as the reference antagonist DPCPX (see Figure 1), allowing alkyl substitutions on the 1 and 3 positions while more bulky, cyclic substituents on the 8 positions proved essential for subtype selectivity. Initial mutagenesis studies indicated an essential role of T270^7.35^ (Met in A_2A_AR and A_2B_AR, Leu in A_3_AR) in the accommodation of substituents at position 8. This was confirmed by two recently solved crystal structures in complex with selective xanthine derivatives [12,13]. Besides revealing T270^7.35^ as a selectivity determinant, the structures showed a slightly widened area as compared to the A_2A_AR in the bottom of the binding pocket, selectively accommodating the substituents at position 1. Finally, the binding pocket of the A_1_AR was shown to be unexpectedly large. All these differences deserve a detailed exploration on the basis of MD and free energy calculations, to understand the specific requirements of ligand binding to this receptor.

**Figure 3:**
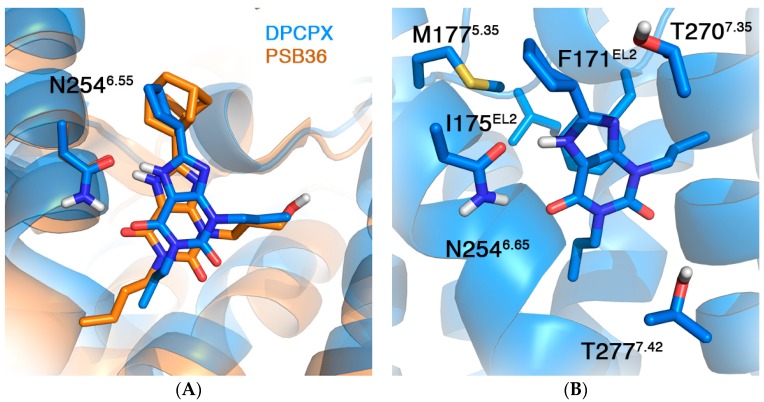
(**A**) Average structure from MD simulations of the DPCPX-A_1_AR complex (blue) superimposed with the crystal structure of the same receptor in complex with PSB-036 (PDB 5N2S); and (**B**) close look in the binding site, highlighting selected residues for in silico mutagenesis studies (see Table 1).

We here apply our in silico mutagenesis approach, described below for the case of A_2A_AR co-crystallized ligands [20,21], to understand the structural requirements of binding of the selective xanthine DPCPX to the A_1_AR. The first stage was to manually dock DPCPX onto the A_1_AR crystal structure 5N2S, based on the structural similarity of the co-crystalized ligand PSB36 and DPCPX (see Figure 3 and Methods). A double hydrogen bond with N254^6.55^ formed the main anchor point between the core scaffold and the receptor, similar to many other crystal structure complexes of A_1_ and A_2A_ ARs.

Although no affinity data is available for DPCPX for the N254A^6.55^ mutant A_1_AR, the fact that many other ligands show severely reduced affinities for N^6.55^ mutant ARs (37 receptor–ligand pairs in the GPCRdb) indicate that this residue is essential for ligand binding. To investigate this hypothesis, we mutated this residue to alanine. As shown in Table 1, the mutation led to unfavorable binding affinities for the mutant receptor. This effect is of similar magnitude as for the F171A^EL2^ mutation, which was shown in literature to result in completely abolished binding [40]. Mutational effects of other residues within the binding site were also correctly reproduced (Table 1). Particularly interesting is T277A^7.41^, the only mutation resulting in favorable binding affinities as compared to WT A_1_AR. This mutation has been thoroughly studied in ligand binding, and is included as a thermostabilizing mutant in the crystal structure 5N2S [13]. Although we correctly capture the favorable effect on ligand binding, there is an overestimation of the effect of about 2 kcal/mol. Therefore, we ran the same mutation on another crystal structure (5UEN) of the A_1_AR, which does not include this thermostabilizing mutation. This time the calculated effect is milder, in agreement with the experimental data (Table 1).

### 2.3. A_2B_AR

**Figure 4:**
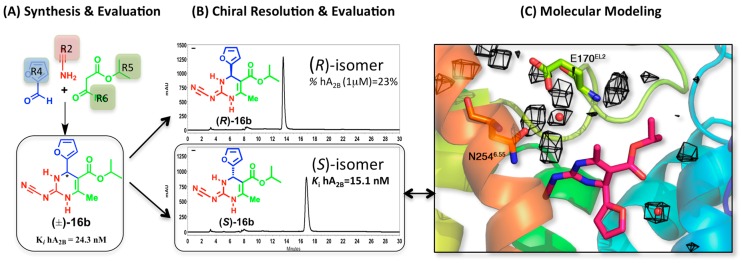
Synthesis and enantiospecific binding characterization of compound **16b** as antagonist of the A_2B_AR: (**A**) the Biginelli multicomponent reaction allows the assembly of the 3,4-dihydropyrimidine scaffold bearing different diversity points; (**B**) the chiral resolution identified the *S* isomer as the one responsible of the biological affinity of the racemic mixture; and (**C**) the molecular modeling confirmed the binding mode previously hypothesized for isomer (*S)*-**16b**. The water-density maps (black mesh) are calculated here by means of MD exploration (grid spacing 1 Å, occupancy > 80%), and are overlaid with the position of structural waters previously considered during the docking of this series (red spheres).

The A_2B_AR is a receptor with low affinity for the natural agonist. It remains silent under physiological conditions to be rapidly activated during chronic highly oxidative stress conditions (e.g., hyperglycemia or mast cell activation). For these reasons, it is an attractive target for inflammatory processes like asthma or colitis, as well as for other diseases like diabetic retinopathy or cancer [1]. However, because of its poor expression levels and low affinity for standard ligands, the pharmacological characterization of this receptor is scarce as compared with the rest of the members of the ARs family. We are consequently engaged in the development of potent and selective antagonists as novel pharmacological tools to aid in this process. The hypothesis underlying this project was the idea that ligands with non-planar, stereodiverse topologies would provide additional selective tools, due to more specific stereoselective ligand-target interactions. We initially designed the 3,4-dihydropyrimidin-2(1*H*)-one scaffold, which is easily assembled via the Biginelli multicomponent reaction, an advantage when it comes to develop series of compounds and evaluate their SAR [29]. The promising affinity of some compounds in the initial series, measured as a racemic mixture, was explored by means of molecular docking on our A_2B_AR homology model. The model revealed an enantiospecific binding orientation, suggesting the potential stereoselectivity of this scaffold and identifying the potentially active isomer (Figure 4). These promising results led to a further exploration of non-planar derived scaffolds, by structural diversification at face 2 of the diazinone scaffold. Three series of analogues were developed, namely 3-deaza[pyridin-2(1*H*)-ones] and bicyclic and tricyclic derivatives fused at positions 2,3 or 5,6 of the heterocyclic framework, identifying selective and potent A_2B_AR ligands (Figure 1) where the hypothesis of the stereoselectivity was maintained [32].

In a later modification of the original scaffold, we created a series of 2-cyanoimino-4-substituted-6-methyl-1,2,3,4-tetrahydro-pyrimidine-5-carboxylates [31]. This time the two enantiomers of the most attractive ligand (**16b**) were separated by chiral HPLC and their absolute configurations established by circular dichroism (Figure 4). The biological evaluation of the two enantiomers demonstrated that the affinity is exclusively due to the *(S)*-**16b** enantiomer, validating the prediction from our molecular modeling studies.

The docking studies of these series of non-planar scaffolds were complemented by MD simulations, which allowed characterizing the role of water molecules in ligand binding. In a first stage, the selected complex of A_2B_AR was equilibrated with our PyMemDyn protocol, as implemented in the GPCR-ModSim web server [44,45] (see Methods). From this analysis, we identified a water molecule that mediated the interaction between the NH in position 1 of the parent 3,4-dihydropyrimidin-2(1*H*)-ones and Glu169^EL2^, and a second water molecule in the region between the alkoxy substituent and the furan/thienyl ring, which is actually equivalent to a conserved water molecule observed in the crystal structures of A_2A_AR with antagonist ZM241385 [23]. These pair of water molecules were maintained in successive docking runs for the expanded series of compounds, verifying their potential role in mediating this inter and intra molecular interactions [29,31,32]. We here provide further support for the existence of these structural water molecules, by means of an MD exploration of the solvent in the binding site (see methods). The idea is that the structural water molecules, which might favor the binding of ligands, is reflected in a higher occupancy during the MD trajectories, as we recently showed for the A_3_AR (see below and reference [30]). Figure 4 shows the calculated water density maps at 80% occupancy, supporting the position of the two water molecules discussed above, mediating interactions between the ligand and E170^EL2^ as well as the intramolecular interaction between the 2-furan and esther groups of the ligand.

### 2.4. A_3_AR

The identification of A_3_AR chemical modulators would help in the development of novel drugs for the pathologies in which this receptor is involved, such as glaucoma, inflammation, asthma and COPD, as well as several types of cancer. The medicinal chemistry developed around this receptor also responds to growing demand for pharmacological tools to study the (pato)physiology of the A_3_AR, and includes the development of agonists and antagonists, as well as radiolabeled versions of these [46,47].

**Figure 5:**
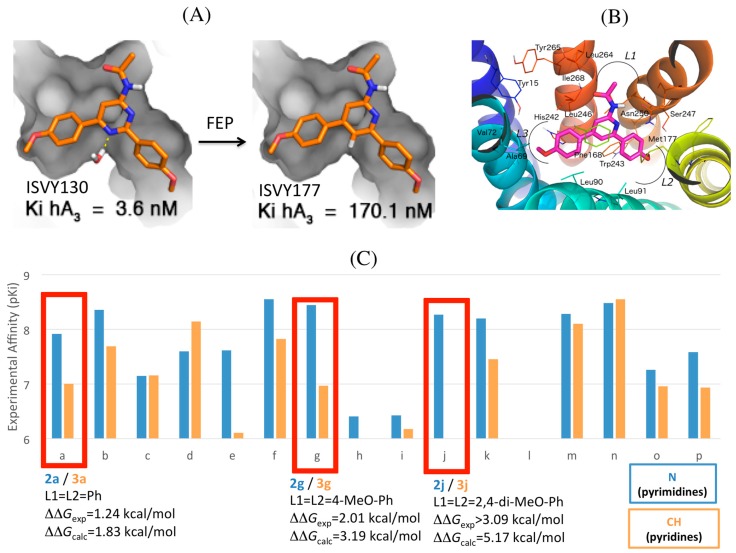
(**A**) Effect of the bioisosteric replacement of pyrimidine (left) by pyridine (right), showing the transformation of N into CH simulated by FEP. The increased affinity in the pyrimidine could be explained by the stabilization of a hydration site as part of a water-network in the binding cavity; (**B**) Binding orientation, common to the two series, is shown for pyridine **ISVY177**; (**C**) Experimental change in binding affinities for each pair of compounds between the two series, with correlation between the corresponding experimental and calculated free energy differences indicated for three selected pairs of compounds (red boxes).

Our initial efforts in this area resulted in the report of several potent and selective antagonists with a monocyclic pyrimidine scaffold (compound ISVY130, Figure 1) [24]. The pyrimidine core, being part of the heterocyclic moiety of the endogenous adenosine, is a recurrent substructural motif within bi- and tricyclic AR antagonists. The functionalized pyrimidine template could be decorated in a divergent and parallel fashion with one alkilamino substituent (L1) and two symmetric aromatic substitution points (L2 and L3, see Figure 5), by means of the well-established Suzuki–Miyaura cross-coupling reaction to a collection of commercially available boronic acids. The initial series of structures combined structural simplicity with a low molecular weight (MW < 350), allowing for optimization during the subsequent hit-to-lead process [24]. This process was assessed by systematic docking on our A_3_AR homology model and the consensus binding mode obtained featured interactions with well-conserved residues in the ARs: the double hydrogen bond of the key residue Asn250^6.55^ with both the exocyclic amido group and the closest nitrogen in the pyrimidine scaffold, and a π-stacking interaction with Phe168^EL2^ complemented by hydrophobic interactions with Leu246^6.51^ (Figure 5). In addition, this binding pose identified selectivity hotspots on the extracellular area around the L1 region: Leu^7.35^ in TM7 (Thr/Met/Met on A_1_/A_2A_/A_2B_, respectively) Ile^6.58^ (Val on the remaining ARs) and Val^5.30^ in EL2 (otherwise Glu in the family). The binding mode and interactions identified were later supported by a crystal structure of the A_2A_AR in complex with another monocyclic scaffold, i.e., 1,2,4-triazine, also bearing aryl substitutions [34]. The biological superposition obtained from the consensus docking was the starting point for a 3D-QSAR model with the second generation of GRid INdependent Descriptors (GRIND-2), used to rationalize and further optimize the three points of diversity of this scaffold. The first model was built on the initial series of 62 compounds, and showed excellent correlation with the experimental data (r^2^ = 0.86) and good predictive power (q^2^ = 0.62). Based on this model, we investigated the role on ligand binding of different methoxyphenyl fragments of the L2/L3 diaryl substituents of the 4-amidopyrimidine scaffold. The increased selectivity of these series confirmed a second A_3_AR selective hotspot created by serines at positions 6.52 (His in the other ARs) and 5.42 (Asn in the other ARs), located deeper at the TM5-TM6 interface, which create a cavity that accommodates the *meta*- and/or *para*-methoxy groups of the most potent and selective compounds [25].

The exploration of the pyrimidine scaffold included the characterization of the two isomeric series, i.e., 2-amidopyrimidines and 4-amidopyrimidines, differing in the position of the second nitrogen (N1) in the pyrimidine ring. The 3D-QSAR model suggested a role of this nitrogen in the affinity of these compounds, presumably by interaction with a potentially conserved structural water molecule within ARs linked to Ser271^7.42^, for which evidence in the hA_3_AR homology model was suggested by a GRID analysis with a water probe [24]. To further explore the complex effect of a “necessary nitrogen” [48] in the structure–activity (SAR) of these heterocycles, we designed a new series of bioisosteres derived from the 4-acetamidopyrimidine scaffold, i.e., lacking this nitrogen atom [30] (Figure 5). The most potent pyridines obtained displayed affinity values in the low nanomolar range, comparable to the best pyrimidines of the former series, maintaining the excellent selectivity profile toward the remaining ARs. The affinity data of the two series remained within the same nanomolar range, which confirmed our design of the bioisosteric replacement. However, a closer look into the data revealed that the nitrogen replacement by CH led to a small to moderate decrease in binding affinity in most compounds within the pyridine series. This would be indeed in line with our previous hypothesis that the second nitrogen would stabilize a water molecule (Figure 5) [24], which in analogy with the latest high resolution crystal structures of the A_2A_AR, could be part of a water network in the deep cavity of the binding site. A comparative MD sampling of the solvent in the two situations (i.e., pyridine and pyrimidine bound states), revealed a specific increase in water densities around the second nitrogen of the pyrimidine, as compared to the A_3_AR-pyridine complex, thus supporting the idea of a water network stabilized by the binding of a molecule bearing the pyrimidine scaffold [30]. We therefore performed FEP simulations, which allowed to quantitatively estimating an understanding the origin of the relative binding affinities between analogous pairs of pyridine/pyrimidine compounds. The calculations, which essentially consisted of the N→CH perturbation in the ring, were in remarkable agreement with the experimental data, reproducing the small effect (8 fold loss in binding affinity) in the non-selective diphenyl substituted pair of compounds, as well as the moderate (30 fold) loss of affinity observed for the most potent compound in the parent pyrimidine series (4-metoxyphenyl derivative, ISVY130, and the corresponding pyridine ISVY177, see Figure 5) [30]. An extreme case was the complete loss in affinity observed for the 2,4-dimethoxyphenyl derivative, which in our models allowed us to determine the most probable binding orientation based on FEP calculations. The 2,4-dimethoxy substituted series could theoretically be accommodated in the binding pocket in four different ways, i.e., depending on the “upwards” or “downwards” orientation of each of the two 2-methoxy substituents, and we ranked each possibility by running parallel FEP calculations on each of them. The results (Figure 5) showed that only the two poses that accommodate the 2-methoxy on L2 looking upwards, i.e., making an additional interaction with Asn250^6.55^, could reproduce the loss in affinity due to the pyrimidine-pyridine perturbation, illustrating the utility of FEP protocols to identify the correct binding mode.

## 3. Discussion

Adenosine receptors have emerged as one of the most extensively characterized families of GPCRs. Efforts of the academia and the pharmaceutical industry led to a vast amount of accumulated pharmacological, biochemical and structural data, providing a highly detailed understanding of ligand binding and receptor function. Together with the tremendous increase of available crystal structures, SBDD on ARs has particularly matured over the past decade. While this is nicely illustrated by the pharmaceutical development of triazine scaffolds as A_2A_AR antagonists [9], we focus here on the understanding of ligand binding and development of novel scaffolds for each of the four ARs, reviewing and updating the results of a decade of collaborative work between our chemical and computational academic labs.

Our integrative approach of efficient synthesis, computational modeling and biological characterization allowed obtaining substantial amounts of novel, chemically simple and selective AR ligands, susceptible of further optimization. In addition, the biological data generated from our new molecules provide the necessary starting point to test our modeling hypotheses, leading to a more complete understanding of ligand binding and receptor activation processes. The antagonists and partial agonists of the A_2A_AR here discussed provide a unique example of understanding ligand functionality on the basis of structural and computational analysis. Our calculations comparing the binding of each molecule type to the active and inactive conformations of the receptor are the first example of a thermodynamic cycle that maps the FEP results to the distinction of pharmacological classes of ligands, going one step further on the recent use of this technology on GPCRs. Future studies will be aimed at the applicability of that approach in the design of novel, non-ribose agonists for ARs. The FEP calculations here reported were set up based on our previously published A_2A_AR antagonist and agonist binding models, and it demonstrates that FEP calculations can be routinely used in drug design once the system is setup and validated, which is the most time-consuming and human-demanding stage.

The recently crystallized A_1_AR inactive structure, combined with the amount of mutagenesis data about antagonist binding to the reference xantine DPCPX, motivated the exploration of the energetic and atomic details of antagonist binding to this receptor here reported, using our “in silico” mutagenesis approach. Our results show excellent agreement with experimental data, giving insights in the determinants of high affinity ligand binding to this receptor and providing us with a new model for future ligand design for this receptor. This approach allows the prediction of the effects of mutations on ligand binding in a systematic and routinely applicable fashion, which is very attractive for the molecular characterization of ligand selectivity between closely related receptors. As we show here with the recent case of the A_1_AR, and previously with the A_2A_AR [20,21], the highest potential of this technique is in combination with experimental mutagenesis and crystal structures, but that can easily be extended to other GPCR systems.

For the remaining ARs (A_2B_ and A_3_ ARs), there is yet no crystal structure available. However, even in these cases we show how homology models with enough quality can be generated to characterize, via molecular simulations, the molecular interactions responsible of changes in ligand binding affinities. This way we could optimize and understand the enantiospecific binding of a novel series of A_2B_AR chiral antagonists, including spurious effects as delicate as water mediated interactions. Water-mediated interactions were also found to be the key element to design and further test the bioisosteric replacement of pyrimidines by pyridines in the A_3_AR, via FEP simulations on our homology-model of this receptor. 

In conclusion, we provide here an overview of our systematic design of new AR ligands, showcasing the applicability of our integrative approach to answer key questions in SBDD on ARs.

## 4. Materials and Methods

### 4.1. Preparation of Starting Receptor Structures

The crystal structures of the human A_1_ (PDB codes 5n2s, with antagonist PBS36 [13], and 5uen, with covalent antagonist DU172 [12]) and A_2A_ ARs (PDB code 4eiy in complex with antagonist ZM241385 [49] and PDB ID 2ydo in complex with the agonist adenosine [50]) were retrieved from the Protein Data Bank, while the corresponding inactive conformations of A_2B_ and A_3_ ARs were generated by homology modeling, using the first crystal structure of A_2A_AR [51] as a template, as described in references [24,29] (sequence ID with the template is 50% for A_2B_AR and 41% for A_3_AR). The initial receptor structures were subject to some refinement as follows before docking and MD simulations: (i) Deletion of the engineered fusion proteins in the crystal structures, reverting the thermostabilyzing mutations (i.e., A_1_AR crystal structure) and building the missing loop segments with Modeller [52]; additional refinement of modeled loops in homology-modeling structures was done using the LoopModeler routine [53]. (ii) Addition of protons and assessment of Asn/Gln/His rotamers and protonation states was performed using Molprobity Web server (http://molprobity.biochem.duke.edu/). In all cases, Asp, Glu, Lys and Arg residues were assigned in their default charged state, and the His residues were modeled as neutral with the proton on Nδ in all cases except for His155^ECL2^ and His250^6.52^ (protonated on Nε), His264^ECL3^ (A_2A_, considered in its positively charged state), and His278 (A_1_, considered in its protonated state as determined for inactive ARs in [44]); (iii) Energy minimization was performed using Schrödinger suite, in the case of homology models [54].

### 4.2. Ligand Docking

The different docking explorations on each AR here reported followed the same scheme: Ligands were build and optimized in the 3D using the Maestro graphical interface and the LigPrep utility from the Schrödinger suite [55]. For the series of enantiospecific ligands, this included considering *R* and the *S* stereoisomer and possible tautomers in parallel dockings. Each ligand was docked 20 times with default (high accuracy) genetic algorithm (GA) search parameters, using the scoring function Chemscore as implemented in GOLD [56] and allowing full flexibility for the ligand, including flipping of amide bonds. The search sphere was centered on the side chain (CD1) of Ile^7.39^ and expanded with a radius of 15 Å, thus ensuring a generous enough search space comprising the orthosteric binding site experimentally determined for adenosine receptors. In the A_2B_AR, two water molecules were considered in the binding cavity, as explored here by MD simulations (see below). In this case, we used the option *toggle trans_spin* 2 in GOLD, meaning that the water contribution is only included if it produces an increase in the predicted scoring and hydrogen bond network is optimized, combined with the *assemble structure* option in GOLD to account for the two rotamers of Asn^6.55^. The criterion for the selection of docking poses was based on a combination of the Chemscore ranking and the population (convergence) of the solutions according to a clustering criterion of 1 Å.

### 4.3. Membrane Insertion, Molecular Dynamics Equilibration and Water Sampling

The receptor structure was inserted into the membrane, solvated and submitted to the MD equilibration protocol PyMemDyn, as implemented in GPCR-ModSim web server [44,45]. Briefly, the protein–ligand complex was inserted in an appropriate orientation in a POPC lipid bilayer soaked with bulk water. The simulation box was created with a hexagonal-prism geometry, which was energy-minimized and carefully equilibrated using periodic boundary conditions (PBC) and the NPT ensemble with the GROMACS simulation package [57]. The MD equilibration scheme included a gradual release of harmonic restraints on protein heavy atoms for 2.5 ns, until only weak backbone restraints were retained on the protein for an additional 2.5 ns. In the case when this equilibration was performed in receptor–ligand complexes, the ligand heavy atoms were also restrained during the first 2.5 ns, with similar progressive decrease in force constants as protein heavy atoms.

For the determination of putative water molecules in the binding site, the final snapshot of the membrane–receptor equilibration of the A_2B_AR was transferred into the MD package Q [58] for MD simulations under spherical boundary conditions (SBC). The MD sampling of the solvent consisted on 10 replicates of 5 ns each, within a sphere of 15 Å around Asn250^6.55^, where all protein atoms were restrained with a 5 kcal/mol/Å^2^ (see next section for detailed parameters of SBC simulations). The occupancy of the solvent in points of a grid of 1 Å mesh was analyzed with the Volmap tool implemented in the VMD software package [59].

### 4.4. Free Energy Perturbation Simulations

The equilibrated binding site of the A_1_ and A_2A_ AR complexes obtained in stage 4.3 were transferred to Q to perform FEP calculations under SBC [58]. In this cases, the SBC was setup with a sphere of 25 Å radius centered on the center of geometry of the ligand, with the following parameters for the MD simulations: protein atoms in the boundary of the sphere (22–25 Å outer shell) are under positional restraints of 20 kcal/mol/Å^2^, while solvent atoms are subject to polarization and radial restrains using the surface constrained all-atom solvent (SCAAS) model to mimic the properties of bulk water at the sphere surface [58,60]. Atoms lying outside the simulation sphere are tightly constrained (200 kcal/mol/Å^2^ force constant) and excluded from the calculation of non-bonded interactions. Long range electrostatics interactions beyond a 10 Å cut off were treated with the local reaction field method [61], except for the atoms undergoing the FEP transformation where no cutoff was applied. Solvent bond and angles were constrained using the SHAKE algorithm [62]. All titratable residues outside the sphere were neutralized and the residues within the sphere manually checked for their most probable state (as detailed in Section 4.1). Residue parameters were translated from the latest version of the OPLSAA/M force field [63], whereas the rest of the parameters for the ligand and lipids were inherited from stage 4.3. The simulation sphere was warmed up during a first equilibration period of 0.61 nanoseconds from 0.1 to 298 K, where an initial restraint of 25 kcal/mol/Å^2^ imposed on all heavy atoms was slowly released. Thereafter, the system was subject to unrestrained MD simulations, starting with a 0.25 nanosecond unbiased equilibration period which is followed by the FEP sampling, where atom transformations occur along a linear lambda (λ) parameter between initial and ending states, equally divided into λ-windows. The sampling consists of 10 replica MD simulations with different initial velocities, each of them with 1 fs time step and a number of 10.000 steps per λ-window. The FEP protocol is divided into separate stages, each of them consisting of the same number λ-windows (for details see [20,21]): initially, the partial charges are removed, a stage that is followed by transformation of the regular van der Waals potentials into smoother soft-core potentials, before the annihilation of the corresponding group of atoms, and finally restoring the partial charges of the final species. For the aminoacid sidechain mutation, these stages (consisting in the current simulations of 21 λ-windows each) are consequently applied to groups of atoms (charge groups), starting from the one with a higher topological distance from the Cα. To fulfill a thermodynamic cycle in these cases, the same sidechain annihilation is simulated in the apo structure of the protein. For the ligand perturbations, the four stages are applied to the group of atoms undergoing change (i.e., CH_2_OH to H), each stage sampled along 51 λ-windows, and parallel transformations of the ligand are done in an equivalent sphere of water (reference state for the thermodynamic cycle). In all reference simulations, the same parameters apply (i.e., sphere size, simulation time, etc.), and the relative binding free energy difference was estimated by solving the thermodynamic cycle utilizing the standard Zwanzig exponential formula [64].

## Figures and Tables

**Table 1:** Mutational effects on DPCPX affinity for A_1_AR. The experimental affinities were retrieved from the GPCRdb and the reference(s) are given in brackets. The binding affinity (pK_D_) was converted to ∆∆G following ∆∆G = RT·ln (K_D_Mutant/K_D_WT).

Mutation	∆∆G (kcal/mol)	
	In Vitro	In Silico
F171A^EL2^ [40]	4.32 ^a^	6.15 ± 0.69
I175A^EL2^ [40]	0.98	0.26 ± 0.39
M177A^5.37^ [40]	1.06	1.84 ± 0.46
N254A^6.55^	ND ^b^	4.14 ± 0.67
T270A^7.34^ [40]	0.46	0.40 ± 0.48
T277A^7.41^ [41,42,43]	−0.32 ± 0.19 ^c^	−2.77 ± 0.55 −0.42 ± 0.54 ^d^

^a^ No detectable binding, the value represents the detection threshold of the experiment; ^b^ No experimental value determined in literature; ^c^ An average value and associated s.e.m. were calculated based on the reported values from literature (*n* = 3); ^d^ Calculations performed on the 5UEN crystal structure.
